# Acute effects of virtual reality treadmill training on gait and cognition in older adults: A randomized controlled trial

**DOI:** 10.1371/journal.pone.0276989

**Published:** 2022-11-02

**Authors:** Lisa A. Zukowski, Faisal D. Shaikh, Alexa V. Haggard, Renee N. Hamel

**Affiliations:** 1 Department of Physical Therapy, High Point University, High Point, North Carolina, United States of America; 2 School of Physiotherapy, University of Otago, Dunedin, New Zealand; University of Massachusetts Lowell, UNITED STATES

## Abstract

**Introduction:**

Everyday walking often involves walking with divided attention (i.e., dual-tasking). Exercise interventions for older adults should mimic these simultaneous physical and cognitive demands. This proof-of-concept study had a two-fold purpose: 1) identify acute cognitive and gait benefits of a single session of virtual reality treadmill training (VRTT), relative to conventional treadmill training (CTT), and 2) identify differences between those who reduced dual-task costs (i.e., responders) on gait or cognition and those who did not, after the session.

**Methods:**

Sixty older adults were randomized to complete a single 30-minute session of VRTT (n = 30, 71.2±6.5 years, 22 females) or CTT (n = 30, 72.0±7.7 years, 21 females). Pre- and post-exercise session, participants performed single-task walking, single-task cognitive, and dual-task walking trials while gait and cognition were recorded. Gait variables were gait speed and gait speed variability. Cognition variables were response reaction time, response accuracy, and cognitive throughput. Dual-task effects (DTE) on gait and cognition variables were also calculated.

**Results:**

Post-exercise, there were no group differences (all *p*>0.05). During single- and dual-task trials, both groups walked faster (single-task: *F*(1, 58) = 9.560, *p* = 0.003; dual-task: *F*(1, 58) = 19.228, *p*<0.001), responded more quickly (single-task: *F*(1, 58) = 5.054, *p* = 0.028; dual-task: *F*(1, 58) = 8.543, *p* = 0.005), and reduced cognitive throughput (single-task: *F*(1, 58) = 6.425, *p* = 0.014; dual-task: *F*(1, 58) = 28.152, *p*<0.001). Both groups also exhibited reduced DTE on gait speed (*F*(1, 58) = 8.066, *p* = 0.006), response accuracy (*F*(1, 58) = 4.123, *p* = 0.047), and cognitive throughput (*F*(1, 58) = 6.807, *p* = 0.012). Gait responders and non-responders did not differ (all *p*>0.05), but cognitive responders completed fewer years of education (*t*(58) = 2.114, *p* = 0.039) and better information processing speed (*t*(58) = -2.265, *p* = 0.027) than cognitive non-responders.

**Conclusions:**

The results indicate that both VRTT and CTT may acutely improve gait and cognition. Therefore, older adults will likely benefit from participating in either type of exercise. The study also provides evidence that baseline cognition can impact training effects on DTE on cognition.

## Introduction

Falls in healthy, older adults are a major public health concern as they often result in serious injury and overall decline in quality of life. Falls experienced by older adults frequently occur when walking or when attention is divided between multiple tasks [1], but, perhaps more concerning, the combination of these two scenarios is common. Walking in everyday life frequently involves simultaneous performance of a cognitive task, such as talking to a friend (i.e., dual-tasking) [2]. Thus, individuals are often distracted while walking, further increasing the likelihood of falling [3]. Dual-task walking involves the coordinated allocation of attentional resources to the simultaneous gait and cognitive tasks, involving executive function, attention, and visuospatial ability [4]. However, with typical aging, a breakdown of network processes and connectivity between cortical regions involved in gait and cognitive processes occurs [5, 6]. These changes result in a shift from automatic to more conscious control of gait, thus limiting the attentional resources available to perform simultaneous tasks and resulting in changes to gait parameters that have been shown to increase risk of falling [4, 7]. Indeed, previous research has shown that increasing age is associated with slower gait speed and increased gait speed variability, relative to young adults, and that these gait impairments become even more pronounced during dual-task walking, further increasing the likelihood of falling in real world environments [8–10]. Previous research has also shown that dual-task walking ability mediates the relationship between fear of falling and performance of activities of daily living [11]. Therefore, it is important for older adults to practice walking while distracted, to mimic everyday life and to reduce likelihood of falling before falls start occurring.

Conventional fall prevention exercise interventions for older adults aim to increase physical activity to improve muscle strength, balance, gait speed, and/or coordination [12–15], which are important for the mechanics of gait and dynamic stability, and conventional physical exercise interventions have been shown to be effective in reducing both fall risk and prevalence [13, 14]. However, impaired cognition is another significant and common fall risk factor for typically aging adults [16]. Previous research has provided conflicting conclusions on whether acute treadmill exercise positively impacts the various domains of cognition and even how moderating factors of the intervention, such as intensity and duration of exercise, and of participant characteristics, such as baseline performance and an individual’s cardiorespiratory fitness, impact the acute effects of treadmill exercise on cognitive domains [17–22]. Only a small number of fall prevention, fall risk reduction, and acute treadmill exercise interventions address cognitive impairments that result in reduced ability to perform dual-tasks, such as shifting attention between walking and talking or visual scanning and planning to negotiate environmental hazards, which increase ongoing risk of falling. Therefore, there is a need for fall prevention and fall risk reduction interventions that address performing dual-tasks, especially everyday dual-tasks like walking while talking or making visuospatial judgements.

Interventions that incorporate simultaneous physical and cognitive training start to address this need and have demonstrated improved cognition, improved dual-task walking performance, and reduced fall prevalence and fall risk [23–27]. Additionally, several studies have provided evidence that a single cognitively-demanding exercise session using commercially available interactive games or virtual reality, commonly called exergaming, can result in acute cognitive performance enhancements [28–30]. However, multiple reviews of conventional cognitive-motor dual-task training interventions and cognitively-demanding exergaming interventions have provided evidence that, although these interventions positively impact cognitive function, there is no consistency across studies in terms of which specific cognitive domains are positively impacted [31, 32]. The review authors determined that the intervention length, design, and specificity of training, including the specific cognitive and mobility demands of the intervention, seem to moderate the impact of the intervention on measured gait and cognitive outcomes. Additionally, the majority of these studies only track gait or cognitive outcome measures, making it difficult to determine the total effects of the interventions [23, 24, 26, 28]. One dual-task intervention study that tracked both motor and cognitive outcomes utilized an interactive game-based training program, which targeted balance, accurate stepping, executive function, attention, and memory [33]. This intervention resulted in improved balance, dual-task Timed Up and Go performance, and global cognition. The intervention failed to improve single-task Timed Up and Go performance, though, likely as a result of the intervention utilizing balance and stepping activities, as opposed to walking, due to constraints of the game system. Additionally, the authors tracked dual-task gait performance and global cognition, but they did not track dual-task cognitive performance. Not tracking dual-task cognitive performance leaves open the question of whether dual-task gait was actually improved or if greater attention was shifted to the walking task, to the detriment of dual-task cognitive performance. Even when authors do track cognitive performance during a dual-task walking condition, they often use cognitive tasks like serial subtraction [27], which is a test of executive function but is not an ecologically valid cognitive task for everyday walking [34], or they only track response accuracy as the measure of cognitive performance [35–37], instead of also tracking response reaction time. Neglecting to additionally track response reaction time may obscure some changes in dual-task performance because older adults typically prioritize maintaining accuracy over speed of response [38], which means reaction time is more likely to change after an intervention than response accuracy. Therefore, more work is needed to evaluate interventions that target and track both cognitive and motor components of dual-task walking, especially cognitive and motor components that are relevant and specific to everyday walking.

A cognitively-demanding exercise intervention utilizing semi-immersive virtual reality during simultaneous gait on a treadmill may be especially suitable for simulating the distractions and demands of walking in everyday life. Indeed, semi-immersive virtual reality on a treadmill can be made not only to replicate the physically- and cognitively-demanding interactions with the game that exergaming has demonstrated, but also allows for a much greater range of activities than exergaming systems (i.e., beyond balance and stepping training or interactive cycling and kayaking ergometers), including walking training that mimics everyday walking demands. Because of this increased specificity of training, gains are more likely to transfer to improved ability to perform cognitively-demanding everyday tasks [39], despite the inconclusive results of acute treadmill training observed in past research. Also, importantly, like exergaming, virtual reality training on a treadmill can be more fun and engaging than typical exercise programs [40], which could improve compliance during an intervention and long-term adherence. The benefits of semi-immersive virtual reality exercise training have only recently started to be explored, pointing towards virtual reality training resulting in improvements in gait, cognition, and dual-task walking performance [40–45]. However, the impact of the training on cognitive performance during dual-task walking has not been examined. The impact of virtual reality training on both gait and cognitive components of dual-task walking in older adults, relative to conventional exercise, is thus a novel focus and should be explored.

Therefore, the study aims are twofold. The primary purpose of this single-session proof-of-concept study was to identify the acute cognitive and gait benefits of a single session of semi-immersive virtual reality treadmill training (VRTT), relative to a single session of conventional treadmill training (CTT). We hypothesized that following a single session of VRTT, older adults will demonstrate improvements in reaction time and accuracy on cognitive tasks and exhibit reductions in dual-task costs on gait and cognition relative to pre-training session cognitive and gait performances and that more acute benefits would be observed after the VRTT than after the CTT, based on the many reported benefits of virtual reality training over conventional training and the relevance of the current study’s VRTT to everyday walking [23, 43, 46]. Because previous research has provided evidence that baseline interindividual differences moderate the acute effects of an intervention but the baseline moderating factors have not been explored in a dual-task intervention [18, 22], the secondary purpose of this study was to identify baseline differences between those who reduced their dual-task costs on gait or cognition and those who did not, from pre- to post-training testing. A better understanding of how individual characteristics influence acute dual-task training gains will enable the development of more effective dual-task interventions, designed to mimic everyday demands and reduce fall risk.

## Methods

### Trial design

This proof-of-concept study was a parallel-group, single-blind, stratified randomized controlled trial with 1:1 allocation ratio. The randomization was stratified on gender, to control for established gait speed differences between males and females and the established influence of gender on exercise-related responses [47, 48]. The randomization sequence was computer-generated and created by one of the authors before the start of the study. The same author screened potential participants and then, in the order that they were screened, assigned male and female participants, separately, via a 1:1 allocation ratio and according to the randomization sequence to complete either a VRTT (experimental) or CTT (active control) exercise session. Participants were blinded to their group allocation and were informed at the conclusion of the study whether they were part of the experimental or active control group.

### Participants

Older adults, who were at least 60 years of age and had not fallen in the last 12 months, were recruited from the local community to participate in the study. Other inclusion criteria were the ability to walk continuously for 8 minutes without the assistance of another person, ability to follow a three-step command, no pre-existing neurological disorders, no uncorrected hearing or visual impairments, and no orthopedic problems affecting ability to walk. Participants were recruited without restriction based on age (except ≥60 years), gender, educational level, ethnicity, and socioeconomic status. Enrolling older adults who were not prone to falling, but who would still be at increased risk of falling when performing a visuospatial dual-task [49], ensured that the results were generalizable to a broad population of older adults, with any significant impacts of the intervention likely to be more pronounced in older adults who are prone to falling.

The study was powered to detect a MANOVA main effect of Time effect size of f = 0.20, which is based on the reported effect of a single cognitively-demanding exercise session on a measure of executive function (i.e., verbal fluency) in older adults [28]. Assuming a correlation among repeated measures of 0.5, the total number of participants needed was 52 (α = 0.05, power = 0.80). This is a medium effect size and therefore likely to be clinically meaningful. This study was approved by the Institutional Review Board (protocol number 201802–677), and all participants provided written informed consent before participating in any study procedures.

### Exercise training session

Participants completed a single 30-minute exercise session of either VRTT (experimental) or CTT (active control) exercise on the same day as the baseline testing and experimental procedures. The exercise session (VRTT and CTT) included 30 minutes of activity separated into six, 5-minute sections. Brief resting periods were built into the exercise session, as game settings needed to be set with the initial loading of each of the three different games and needed to be changed between easier and harder versions of each game. Participants were allowed to rest for longer between sections if they desired. All rest periods were in addition to the 30 minutes of exercise activity that each participant completed. Participants were informed at the conclusion of the study whether they were part of the experimental or active control group.

The single VRTT sessions were completed on the GRAIL (Motek Medical, Amsterdam, The Netherlands), a semi-immersive virtual reality system with an instrumented dual-belt treadmill positioned within and integrated with a 180-degree curved projection screen, in the Virtual Reality and Clinical Gait Analysis Laboratory at High Point University. Participants completed two versions, an easier and a harder version, of three different exercise games, for a total of six exercise sections. All three exercise games were selected from the modules included with the system and were run through the D-Flow software (Motek Medical, Amsterdam, The Netherlands). The session started with the Boardwalk game, a self-paced walking module that entailed participants controlling and matching their gait speed to target speeds while ignoring distractions, requiring sustained and selective attention. The VRTT session then progressed to the Italian Alps game, a self-paced walking module that required participants to engage in identifying and gathering specified items and avoiding distracting items and obstacles. This involved modulating gait speed, maintaining dynamic balance while walking and simultaneously moving from side to side on the treadmill, and utilizing quick information processing and planning. Finally, the VRTT session finished with the Step on It game, a set-speed walking module that required participants to correctly step their avatar’s feet into boxes on the screen. Step length, step width, and cadence were constantly changing, requiring participants to modulate their spatiotemporal gait parameters, utilize quick information processing, and maintain sustained attention. For each of the three games, all gait speed targets were based on self-selected gait speed, as measured during overground walking. Degree of difficulty also progressed with each subsequent game. Details of the three games are provided in Table 1. These three games were chosen because of their applicability to the performance of everyday tasks and fall risk.

**Table 1:** The three exercise games performed during the VRTT and CTT sessions. During the CTT sessions, participants did not encounter the visual distractions, as the video and auditory components were turned off. For each game, participants performed an easier and a harder version.

Game	Description
Boardwalk	Self-paced walking game on an ocean boardwalk. Participants control their walking speed by walking more quickly, causing the treadmill belts to accelerate, or more slowly, causing the treadmill belts to decelerate. Participants must match and maintain their walking speed to different target speed ranges, based on percentages of their self-selected overground walking speed. Participants must attend to the speed monitor at the center of the screen, which shows the participant’s current speed and the speed range that they must stay within, while ignoring the distractions in the surrounding ocean and air space. • Easier version: three broad speed ranges (0.5*self-selected– 1.0*self-selected speed) • Harder version: five narrower speed ranges (0.5*self-selected– 1.25*self-selected speed) • Each version of this game was performed for five minutes
Italian Alps	Self-paced walking game in an Italian village street. Participants control their walking speed while also shifting center of mass, depicted by a wooden cart avatar, to walk on the left and right sides of the treadmill. Participants must walk and move around the treadmill while gathering needed pizza ingredients to make pizzas and avoid obstacles in the virtual walking path. Participants must modulate gait speed and maintain dynamic balance to gather items and avoid obstacles, while utilizing quick information processing and planning. • Easier version: needed items placed farther away from obstacles (0.5*self-selected– 1.0*self-selected speed) • Harder version: needed items placed immediately before and after obstacles that needed to be avoided (0.5*self-selected– 1.25*self-selected speed) • Each version of this game was performed for five minutes
Step on It	Set-speed walking game on a plane runway. Participants must adjust their step length, step width and cadence to accurately step their avatar’s feet into boxes on the screen. The walking speed is based on self-selected overground walking speed, and the system records baseline step length and step width during a baseline walking period. After the baseline period, step length and width are changed as a percentage of baseline measures each minute to prompt participants to take long and wide steps, long and narrow steps, short and narrow steps, and finally short and wide steps. Participants must modulate their spatiotemporal gait parameters, utilize quick information processing, and attend to the accuracy of their foot placement on the screen. • Easier version: long and wide (110% and 140% of baseline length and width, respectively), long and narrow (120% and 70%), short and narrow (90% and 80%), and short and wide (80% and 150%) steps • Harder version: long and wide (130% and 140% of baseline length and width, respectively), long and narrow (140% and 60%), short and narrow (80% and 90%), and short and wide (70% and 140%) steps • Each version of this game was performed for five minutes

The single CTT sessions were also completed on the GRAIL and required participants to perform the same three exercise games, using the same percentages based on overground self-selected walking speed and baseline step length and width as utilized with the VRTT group, but with the video projection and audio sounds turned off. Instead of interacting with the video and auditory components, a research assistant, who could see the D-flow computer depicting what would have been projected onto the projector screen, instructed participants on how to modulate gait speed, position and spatiotemporal gait parameters on the treadmill as dictated by each game. Thus, the CTT session required participants to perform the same series of motor tasks as the VRTT group at the same training intensity, but without video and auditory components and instead mimicking a session with a physical therapist or a personal trainer. Thus, the critical difference between the groups were the cognitively-demanding interactive nature of the VRTT session relative to conventional exercise.

### Procedures

Participants from both groups completed the same testing and procedures in the Virtual Reality and Clinical Gait Analysis Laboratory at High Point University. At the start of testing, demographic information was collected. Additionally, participants were assessed to establish baseline cognitive abilities, motor abilities, visual acuity, and community participation. Specifically, the Montreal Cognitive Assessment was utilized to assess global cognition [50], the Coding subtest from the Wechsler Adult Intelligence Scale 4^th^ edition was utilized to assess information processing speed [51], the Comprehensive Trail Making Test was utilized to assess focused attention and inhibition of distraction [52], and a computerized version of the Stroop color-word interference test was utilized to assess selective attention and inhibition of habitual response. For motor abilities, the 10-meter Walk Test was utilized to assess self-selected walking speed, the Timed Up and Go was utilized to assess functional mobility [53], the Four Square Step Test was utilized to assess dynamic balance [54], and the 30-second Sit-to-Stand Test was utilized to assess functional lower extremity strength [55]. Visual acuity was assessed utilizing the Snellen Test. Finally, for community participation, the Physical Activity Scale for the Elderly was utilized to assess self-reported physical activity [56], and the Activities-specific Balance Confidence Scale was utilized to assess balance self-efficacy [57].

After baseline assessments, participants completed a series of three tasks both immediately before and immediately after the exercise training session in a quasi-randomized order: walking at a self-selected speed (single-task walking), performing a cognitive task while seated (single-task cognitive), and performing a cognitive task while simultaneously walking at a self-selected speed (dual-task walking). The order of the three tasks was quasi-randomized because the dual-task walking trial was always performed prior to the single-task walking trial. In previous studies, individuals typically walked faster during a single-task walking trial performed after a dual-task walking trial, as compared to during a single-task walking trial performed before a dual-task walking trial [58, 59]. Standardizing the dual-task walking trial to occur prior to the single-task walking trial ensured that the effect of task order on gait speed and the dual-task effect on gait speed was consistent across participants. Task order was maintained for each participant, from pre- to post-exercise session. To determine the acute effects of the exercising training session, post-testing was completed within 15 minutes after the exercise session to ensure that those acute effects did not dissipate [19]. All three of the trial conditions were one minute in duration.

During the walking trials, participants walked continuously for one minute along a 20-meter path that was free of obstructions and allowed for plenty of room to turn at either end of the path. Walking continuously for one minute resulted in participants taking, on average 42 (SD 5.2) strides, which is well above the 50 steps (i.e., 25 strides) suggested as the optimal number of steps necessary for reliable estimation of gait variability [60]. Before each walking trial, participants were instructed to walk at their preferred, comfortable speed and, before the dual-task walking trial, participants were not given specific prioritization instructions. Gait data were recorded using a wireless 6-sensor inertial measurement unit system (128 Hz, Opal Sensors, APDM Inc., Portland, OR). Sensors were placed on the dorsum of each foot, on the lumbosacral junction, on the sternum, and on each wrist. Stride velocity was calculated for each steady-state stride using the validated Mobility Lab software (APDM Inc.) [61], which excludes gait startup, gait stopping, and turns. Gait data were then further post-processed in a custom Matlab (MathWorks, Natick, MA) program to ensure that only one minute of steady-state gait data were included in the analysis, and to calculate average gait speed (m/s) and gait speed coefficient of variation (cv, %) for each trial. The primary gait outcome variable was single-task and dual-task gait speed, and the secondary gait outcome variable was gait speed cv.

The cognitive test used in this study during the single-task cognitive and dual-task walking trials was the auditory “clock task”, which is a visuospatial reaction time test [62]. This test was chosen because of the applicability of visuospatial decision-making during walking in everyday life to avoid obstacles and plan a walking path. In this test, participants heard a series of numerical times and were asked to say “yes” if both clock hands were on the same vertical half of the clock and “no” if the clock hands were on different vertical halves of the clock for each time. Prior to testing, participants performed two practice blocks of 30 stimuli to become familiarized to the task. The stimuli were produced using DirectRT software (v2016, Empirisoft Corporation, New York, NY) and delivered through wireless headphones (H800, Logitech, Newark, CA) that also recorded verbal responses via a microphone in the headset. Participants performed the clock task for one minute while seated in a quiet room during the single-task cognitive trial and for the entire minute that they were walking during the dual-task walking trial. Before each cognitive trial, participants were instructed to respond as quickly and accurately as possible to each stimuli, and, during the dual-task walking trial, participants were not given specific prioritization instructions. Response reaction time (ms) was recorded and response accuracy (%) was calculated. To account for the speed-accuracy trade-off, a cognitive throughput was calculated as response accuracy divided by average response reaction time and multiplied by a scaling factor (600 = 60,000/100, to convert the percentage to a number and reaction time from milliseconds to minutes), in units of number of correct responses per minute of responding [63]. A higher cognitive throughput value, a combination of greater accuracy and/or faster reaction time, therefore, indicates better performance. The primary cognitive outcome variable was single-task and dual-task cognitive throughput, and the secondary cognitive outcome variables were cognitive response reaction time and cognitive response accuracy.

To account for changes from single- to dual-task performances, dual-task effects on gait and cognition were calculated. The dual-task effect on gait speed (DTE_GS_), on gait speed cv (DTE_GSCV_), on cognitive response reaction time (DTE_RT_), on cognitive response accuracy (DTE_ACC_), and on cognitive throughput (DTE_CT_) were calculated using gait speed (GS), gait speed cv (GSCV), cognitive response reaction time (RT), cognitive response accuracy (ACC), and cognitive throughput (CT) calculated from the single- and dual-task trial conditions as follows [64]:

$${\text{DTE}}_{\text{GS}}=\frac{\left(dual-task\;GS-single-task\;GS\right)}{single-task\;GS}\times 100\%$$

$${\text{DTE}}_{\text{GSCV}}=\frac{-(dual-task\;GSCV-single-task\;GSCV)}{single-task\;GSCV}\times 100\%$$

$${\text{DTE}}_{\text{RT}}=\frac{-(dual-task\;RT-single-task\;RT)}{single-task\;RT}\times 100\%$$

$${\text{DTE}}_{\text{ACC}}=\frac{\left(dual-task\;ACC-single-task\;ACC\right)}{single-task\;ACC}\times 100\%$$

$${\text{DTE}}_{\text{CT}}=\frac{\left(dual-task\;CT-single-task\;CT\right)}{single-task\;CT}\times 100\%$$

For all five DTE values, a negative value indicates a dual-task cost on performance (i.e., slower gait speed, more variable gait speed, slower responses, less accurate responses, or less accurate/slower responses), relative to single-task performance. A positive value indicates a dual-task benefit on performance, relative to single-task performance. The DTE outcome variables were DTE_GS_, DTE_GSCV_, DTE_RT_, DTE_ACC_, and DTE_CT_, which were all secondary outcome variables.

### Statistical analysis

Independent samples t-tests were used to compare demographics, baseline cognitive abilities, baseline motor abilities, visual acuity, and community participation in the VRTT and CTT groups. The dependent variables were gait speed, gait speed cv, cognitive response reaction time, cognitive response accuracy, cognitive throughput, DTE_GS_, DTE_GSCV_, DTE_RT_, DTE_ACC_, and DTE_CT_. Multivariate repeated measures ANOVAs were utilized to compare the dependent gait and cognitive task variables before and after the exercise session for each group (Time x Group). The assumptions to run a MANOVA were explored. First, each observation was randomly and independently sampled from the population. Second, each dependent variable has an interval measurement. Third, the multivariate normality assumption was not met. Instead, the dependent variables were not normally distributed, which is not atypical for gait and cognitive performance data. Outliers in the data were explored, and it was determined that the two gait variable outliers were different from the three cognitive variable outliers, and none of those individuals were consistent outliers for all variables. Because MANOVA is robust to violations of multivariate normality provided there aren’t many outliers and because all of these outliers were determined to be real performances, these five individuals were not removed from the analysis. Fourth, Box’s M test of equality of covariance matrices was significant, so Pillai’s Trace Test was used to examine significance of the omnibus effect. Finally, the dependent variables are appropriately correlated with each other, as evidenced by the majority of the dependent variables exhibiting correlation coefficients between 0.30 and 0.89. There were two correlation coefficients observed within the cognitive variables that were greater than 0.9, suggesting multicollinearity could be a problem. To investigate the impact of these variables, they were removed from the analysis, and the MANOVA was rerun. Other than the loss of one significant pairwise comparison involving one of the variables that had been removed, the results of the reduced cognitive variable MANOVA were not different from the full cognitive variable MANOVA. Therefore, the two highly correlated variables were determined to not be problematic for the analysis, and the full set of cognitive variables were included in the reported MANOVA. Independent samples t-tests were used to compare demographics, baseline cognitive abilities, baseline motor abilities, visual acuity, and community participation between those who reduced their DTE_GS_ and DTE_CT_ (responders), separately, and those who did not (non-responders), from pre- to post-training testing. Difference scores from pre- to post-training testing DTE_GS_ and DTE_CT_ were used to define gait and cognitive responders and non-responders [65]. All t-tests and MANOVAs were performed using SPSS 27 and α = 0.05.

## Results

**Fig 1:**
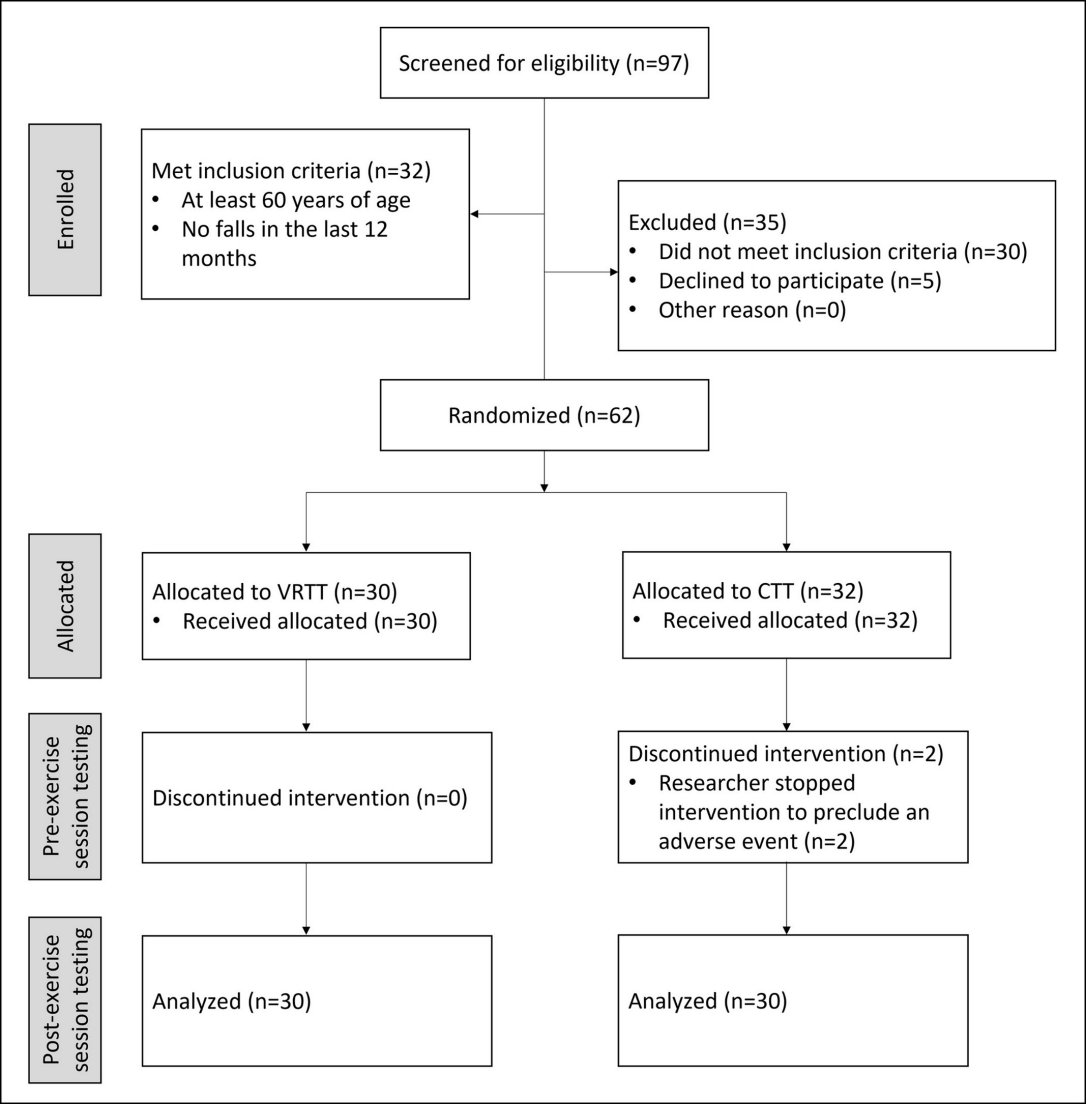
Participant flow diagram.

Sixty-two older adults participated in the study, including 30 in the VRTT group and 32 in the CTT group. After the start of the exercise session, two of the CTT participants were deemed at risk of suffering an adverse event and were prevented from completing the exercise session by one of the researchers. Thus, these two CTT participants did not undergo the post-exercise session testing procedures. Therefore, the analyzed sample included 30 older adults in the VRTT group and 30 older adults in the CTT group, who completed all of the exercise session and testing procedures (Fig 1). There were no significant differences between the groups in terms of demographics, baseline cognitive abilities, baseline motor abilities, visual acuity, or community participation (all p>0.05, Table 2). Therefore, these groups were suitable for comparison.

**Table 2:** Characteristics of participants in the VRTT and CTT groups.

	VRTT (n = 30)	CTT (n = 30)	p-value
**Demographic Characteristics**			
Age (years)	71.2±6.5	72.0±7.7	p = 0.64
Gender	8 males, 22 females	9 males, 21 females	p = 0.78
Years of Education	15.9±3.1	16.5±3.6	p = 0.49
**Cognitive Assessments**			
Montreal Cognitive Assessment (max. 30)	25 (23–27)	26.0 (23–27)	p = 0.72
WAIS IV Coding (scaled score, max 19)	12 (10–14)	11 (10–12)	p = 0.37
Comprehensive Trail Making Test (%)	50 (21–77)	60 (21–73)	p = 0.89
Stroop Color-word Test Interference Reaction Time (ms)	347.7±116.0	338.3±104.2	p = 0.74
**Functional Mobility Assessments**			
10 Meter Walk Test (m/s)	1.4 (1.2–1.5)	1.3 (1.2–1.4)	p = 0.97
Timed Up and Go (sec)	8.9 (7.8–9.8)	8.9 (8.3–9.8)	p = 0.76
Four Square Step Test (sec)	9.6±2.0	10.2±2.3	p = 0.35
30 Second Sit-to-Stand (#)	13 (11–15)	13 (11–14)	p = 0.37
**Visual Acuity**			
Mini Snellen Test (with corrective lenses, normal is 20/20)	20/25 (20/40–20/16)	20/30 (20/40–20/20)	p = 0.43
**Community Participation and Self-Efficacy**			
Physical Activity Scale for the Elderly	174.1±69.9	161.2±76.4	p = 0.50
Activities-Specific Balance Confidence Scale (max. 100%)	95.3 (84.4–98.1)	92.2 (85.6–95.0)	p = 0.59

The p-values represent the results of independent samples t-tests. Values are Mean±SD or Median(IQR).

For the gait variables (i.e., gait speed, gait speed cv, DTE_GS_, and DTE_GSCV_), the results of the repeated measures MANOVA indicated that there was a main effect of Time (*F*(6, 53) = 4.825, *p* < 0.001, $\eta_{p}^{2}$ = 0.353) because the effect of time on single-task gait speed (*F*(1, 58) = 9.560, *p* = 0.003, $\eta_{p}^{2}$ = 0.142), dual-task gait speed (*F*(1, 58) = 19.228, *p* < 0.001, $\eta_{p}^{2}$ = 0.249), and DTE_GS_ (*F*(1, 58) = 8.066, *p* = 0.006, $\eta_{p}^{2}$ = 0.122) was significant (Table 3). Pairwise comparisons indicated that single-task gait speed was 0.04 m/s faster, dual-task gait speed was 0.09 m/s faster, and the DTE_GS_ was reduced by 3.94% during post-exercise testing relative to pre-exercise testing. There was no main effect of Group (*F*(6, 53) = 1.750, *p* = 0.128, $\eta_{p}^{2}$ = 0.165) or a significant Group x Time interaction effect (*F*(6, 53) = 0.221, *p* = 0.968, $\eta_{p}^{2}$ = 0.024).

**Table 3:** Average gait and cognitive performance for VRTT and CTT participants during single- and dual-task performances before and after the exercise session.

	VRTT (n = 30)		CTT (n = 30)	
	Pre-exercise	Post-exercise	Pre-exercise	Post-exercise
**Gait Speed (m/s)**				
Single-Task Walking	**1.28 ± 0.03**	1.33 ± 0.03*	**1.25 ± 0.03**	1.28 ± 0.03*
Dual-Task Walking	**1.10 ± 0.04**	1.18 ± 0.04*	**1.06 ± 0.04**	1.16 ±0.04*
**Gait Speed CV (%)**				
Single-Task Walking	4.06 ± 0.17	3.99 ± 0.21	3.66 ± 0.17	3.62 ± 0.21
Dual-Task Walking	6.78 ± 0.45	6.12 ± 0.54	6.26 ± 0.45	5.83 ± 0.54
**Dual-Task Effect (%)**				
DTE_GS_ (%)	**-14.95 ± 1.88**	-11.68 ± 1.66*	**-14.69 ± 1.88**	-10.09 ±1.66*
DTE_GSCV_ (%)	-72.93 ± 11.41	-61.33 ± 12.26	-71.17 ± 11.41	-60.95 ± 12.26
**Cognitive Response Reaction Time (ms)**				
Single-Task Seated	**1911.5 ± 107.6**	1803.5 ± 132.6*	**2080.6 ± 107.6**	2026.6 ± 132.6*
Dual-Task Walking	**2203.5 ± 226.9**	2047.2 ± 113.9*	**2608.0 ±226.9**	2143.5 ± 113.9*
**Cognitive Response Accuracy (%)**				
Single-Task Seated	93.6 ± 1.7	91.0 ± 2.0	94.5 ± 1.7	95.5 ± 2.0
Dual-Task Walking	87.7 ± 2.2	90.7 ± 1.9	93.6 ± 2.2	95.5 ± 1.9
**Cognitive Throughput (correct responses per minute)**				
Single-Task Seated	**30.69 ± 1.33**	31.62 ± 1.57*	**29.27 ± 1.33**	31.51 ± 1.57*
Dual-Task Walking	**24.85 ± 1.33**	27.92 ± 1.33*	**25.19 ± 1.33**	28.63 ± 1.33*
**Dual-Task Effect (%)**				
DTE_RT_ (%)	-16.35 ± 3.65	-14.68 ± 3.56	-20.30 ± 3.65	-9.32 ± 3.56
DTE_ACC_ (%)	**-6.18 ± 1.95**	0.33 ± 1.87*	**-0.71 ± 1.95**	0.60 ± 1.87*
DTE_CT_ (%)	**-18.11 ± 2.80**	-9.76 ± 3.30*	**-14.82 ± 2.80**	-6.11 ± 3.30*

* Indicates a significant main effect of Time at the α = 0.05 level

Values are Mean±SE.

For the cognitive variables (i.e., cognitive reaction time, cognitive response accuracy, cognitive throughput, DTE_RT_, DTE_ACC_, and DTE_CT_), the results of the repeated measures MANOVA indicated that there was a main effect of Time (*F*(9, 50) = 4.990, *p* < 0.001, $\eta_{p}^{2}$ = 0.473) because the effect of time on single-task cognitive throughput (*F*(1, 58) = 6.425, *p* = 0.014, $\eta_{p}^{2}$ = 0.100), dual-task cognitive throughput (*F*(1, 58) = 28.152, *p* < 0.001, $\eta_{p}^{2}$ = 0.327), DTE_ACC_ (*F*(1, 58) = 4.123, *p* = 0.047, $\eta_{p}^{2}$ = 0.066), single-task reaction time (*F*(1, 58) = 5.054, *p* = 0.028, $\eta_{p}^{2}$ = 0.080), dual-task reaction time (*F*(1, 58) = 8.543, *p* = 0.005, $\eta_{p}^{2}$ = 0.128), and DTE_CT_ (*F*(1, 58) = 6.807, *p* = 0.012, $\eta_{p}^{2}$ = 0.105) was significant (Table 3). Pairwise comparisons indicated that participants gave 1.59 more correct answers per minute during the single-task condition, gave 3.26 more correct answers during the dual-task condition, responded 81.1 ms more quickly during the single-task condition, responded 310.4 ms more quickly during the dual-task condition, exhibited a 3.91% smaller DTE_ACC_, and exhibited a 8.53% smaller DTE_CT_ during post- releative to pre-exercise session testing. There was no main effect of Group (*F*(9, 50) = 0.974, *p* = 0.473, $\eta_{p}^{2}$ = 0.149) or a significant Group x Time interaction effect (*F*(9, 50) = 0.815, *p* = 0.605, $\eta_{p}^{2}$ = 0.128).

In comparing those who reduced their DTE_GS_ from pre- to post-exercise session testing (gait responders), relative to those who increased their DTE_GS_ (gait non-responders), across both exercise interventions, there were no significant differences between the 38 gait responders and 22 non-responders (all *p* > 0.05). In comparing those who reduced their DTE_CT_ from pre- to post-exercise session testing (cognitive responders), relative to those who increased their DTE_CT_ (cognitive non-responders), there were differences between the 36 cognitive responders and 24 cognitive non-responders in terms of years of education completed (*t*(58) = 2.114, *p* = 0.039) and WAIS IV Coding scaled scores (*t*(58) = -2.265, *p* = 0.027). The cognitive responders completed an average of 1.8 fewer years of formal education than non-responders (15.5 and 17.3 years, respectively) and earned a Coding score that was 1.3 scaled points higher than the non-responders (11.9 and 10.6, respectively).

## Discussion

The primary purpose of this study was to identify the acute cognitive and gait benefits of a single session of VRTT, relative to a single session of CTT. There were no differences in the improvements between the VRTT and CTT groups. Instead, in partial support of our hypothesis, both VRTT and CTT groups improved performances from pre- to post-exercise session. In terms of gait speed, they walked 0.04 m/s faster during the single-task walking condition and 0.09 m/s faster during the dual-task walking condition after the exercise session, which can both be considered meaningful changes in gait speed as they are consistent with reported small and moderate gait speed effects, respectively, for older adults [66]. Because participants in this study increased their dual-task gait speed even more than their single-task gait speed, DTE_GS_ was also reduced, which is associated with decreasing risk of falling. These gait improvements are consistent with previous research that demonstrated faster dual-task gait speed in individuals with Parkinson’s disease and healthy older adults after a single-session of split-belt or tied-belt treadmill training [21].

In terms of cognitive performance, both groups gave 1.59 and 3.26 more correct answers per minute and responded 81.1 ms and 310.4 ms more quickly during single- and dual-task conditions, respectively, after the single exercise session. Although there are no published meaningful changes for cognitive throughput or for reaction time and accuracy of the clock task, the pre- to post-exercise session changes in cognitive throughput can be considered meaningful because they exceed the standard error of the mean for single-task (SEM = 0.94) and dual-task (SEM = 0.93) cognitive throughput before the exercise session for the combined VRTT and CTT groups. Because the dual-task improvements in cognitive throughput were greater than the single-task improvements, both groups exhibited reduced dual-task costs on accuracy and cognitive throughput. These improvements in cognitive performance are consistent with those previously reported in the literature, demonstrating improvements in single-task reaction time in older adults after an acute bout of aerobic exercise and in dual-task reaction time and response speed/accuracy tradeoff after an acute bout of exercise involving a more complex walking task [19, 21, 67].

These gait and cognition results likely provide evidence that both the VRTT and CTT exercise interventions acutely improved performance of both a visuospatial reaction time task and gait speed in single-task and dual-task conditions. Additionally, these results, especially the reduction in dual-task costs on gait speed and cognitive throughput, likely provide evidence that these interventions can acutely improve overall dual-task performance and rule out a simple shift of attention to the walking task, to the detriment of the cognitive task being simultaneously performed. These positive effects are supported by previous research that determined acute bouts of exercise could improve interference control, improving dual-task performance because of either an augmentation of overall attentional capacity or an increase in automaticity of the motor task, allowing for greater attentional resources to be devoted to the cognitive task [21, 22]. These acute benefits could also likely be transformed into more permanent improvements in gait and cognition [23] if the VRTT and CTT training were administered in a multi-week exercise program. Because of the everyday importance of making timely decisions based on visuospatial information in the environment while simultaneously walking, these results are significant and indicate the potential utility of treadmill training interventions that involve complex, everyday walking demands. Although, the study design does not rule out the possibility that the acute improvements in gait and cognition were the result of test-retest effects unrelated to the interventions, the likelihood of this occurrence is limited by practice of the tasks before testing started. Specifically, participants performed multiple practice trials of the cognitive task to become familiar to and as fast/accurate as possible at performing the task before completing the pre-exercise session single-task cognitive trial. Additionally, participants performed the 10-meter Walk Test at a self-selected speed along the same 20-meter path used for the single- and dual-task walking trials, prior to completing either the single- or dual-task walking trials.

As previously mentioned, there were no differences in the improvements between the VRTT and CTT groups. This study was designed to be a proof-of-concept study, so it was powered to detect a within-group effect and not to detect between-group or within-between interaction effects. However, a post-hoc power analysis using the Time x Group Pillai’s Trace value (P = 0.128) for the cognitive variables provides evidence that the study was adequately powered to detect a Time x Group interaction effect (post-hoc power = 0.83), if such an interaction effect had existed. Therefore, the study results are valid, despite the relatively small sample size, and the lack of differences between the groups is unexpected, given the many reported benefits of exergaming and virtual reality training, over conventional training [23, 27, 43, 46, 68]. There are many potential reasons why no differences between the groups post-exercise session may have been observed, though. First, this study was again meant to be a proof-of-concept study, and thus older adults only participated in a single session of exercise training. While the acute effects of the two exercise sessions did not differ, if the same two protocols were expanded to be delivered multiple times a week for greater than eight weeks as progressively challenging exercise programs, then the effects of the two programs may have differentiated themselves more, especially in terms of dual-task performance of gait and cognitive tasks. Indeed, a meta-analysis focused on the impact of duration on the effectiveness of an exercise intervention has determined that at least eight weeks of continuous training is often necessary to differentiate the relative benefits of two different types of exercise [69].

Second, the control group in this study, the CTT group, was an active control, who went through the same physical training program as the VRTT group. The only difference between the experiences of the two groups, therefore, was that, in the CTT group, a research assistant instructed participants on how to change their movements (i.e., modulate their speed, move from side to side, and change their step length and width), while, in the VRTT group, participants had to interact with the screen to understand what they were supposed to do and to make the same changes. Thus, because it required participants to interact with instructions from a research assistant, the CTT training still may have been cognitively demanding and more of an open task, similar to the VRTT training, even without the extra interaction with the screen. Previous research has also provided evidence that an acute exercise session can result in transient improvements in cognitive performance because of physiological changes, such as heart rate, brain-derived neurotropic factor, and serotonin in response to exercise, that have implications for cognitive function [18]. More specifically, the performance of physically demanding open tasks may result in trophic factors being synthesized and secreted during these tasks, stimulating neuroplasticity as a result of the differentiation and proliferation of neurons and potentially increasing the functioning of specific brain circuits related to cognition [70, 71]. Thus, both groups may have improved their cognitive performances because of the physical demands of the training protocols, irrespective of the cognitive demands of the two protocols. Previous research has similarly failed to identify differences between a virtual reality/exergaming intervention and an active control group in shorter duration interventions that were a single session or 6–8 weeks in length [28, 40, 41, 72]. A longer duration study may have elucidated differences between the two interventions, as previously mentioned.

Third, some participants caught on to interacting with the screen more quickly than others, so not everyone had the same experience with the VRTT session. While many older adults in the VRTT group caught on very quickly and seemed to enjoy the game-like aspect of the training protocol, others struggled to understand what they were supposed to do and required a lot of verbal instructions from the research assistant in order to achieve the goals of each training game. In other words, not all VRTT participants experienced the same amount of visual interaction training, and some of the VRTT participants may have had a more similar experience to the CTT group (i.e., frequently interacting with and receiving a lot of verbal instructions from the research assistant) than to the rest of the VRTT group. Monteiro-Junior et al. [28] similarly reported that a single session may not have been enough time for all of their older adult participants to understand the exergaming activities performed. It is possible that some individuals are more inclined to perform and adhere to a virtual reality training program, while others may benefit more from one-on-one interaction with a trainer or physical therapist. Indeed, a recent systematic review addressing the use of virtual reality and exergaming systems at home by older adults reported strong but not always perfect retention (ranging from 70–100%) and adherence (ranging from 63–100%) [73].

Finally, while pre-/post-exercise testing utilized a visuospatial reaction time test and the VTT was designed to target reactions to visuospatial stimuli, subtle dissimilarities between the training and testing task may have limited the transfer of the training effects to the outcome measure, especially in an acute timeframe. Indeed, training is known to lead to highly task-specific learning and does not always lead to learning of more generalized abilities [74]. In other words, because the training focused on making decisions about how to move the body in space in response to visuospatial stimuli, this learning may not have fully transferred to the cognitive task, which required making decisions about the location of clock hands visualized in the mind. The use of a cognitively-demanding motor task as an outcome measure, such as stepping over obstacles, may provide evidence of a larger training effect in the VTT, relative to the CTT, group.

**Fig 2:**
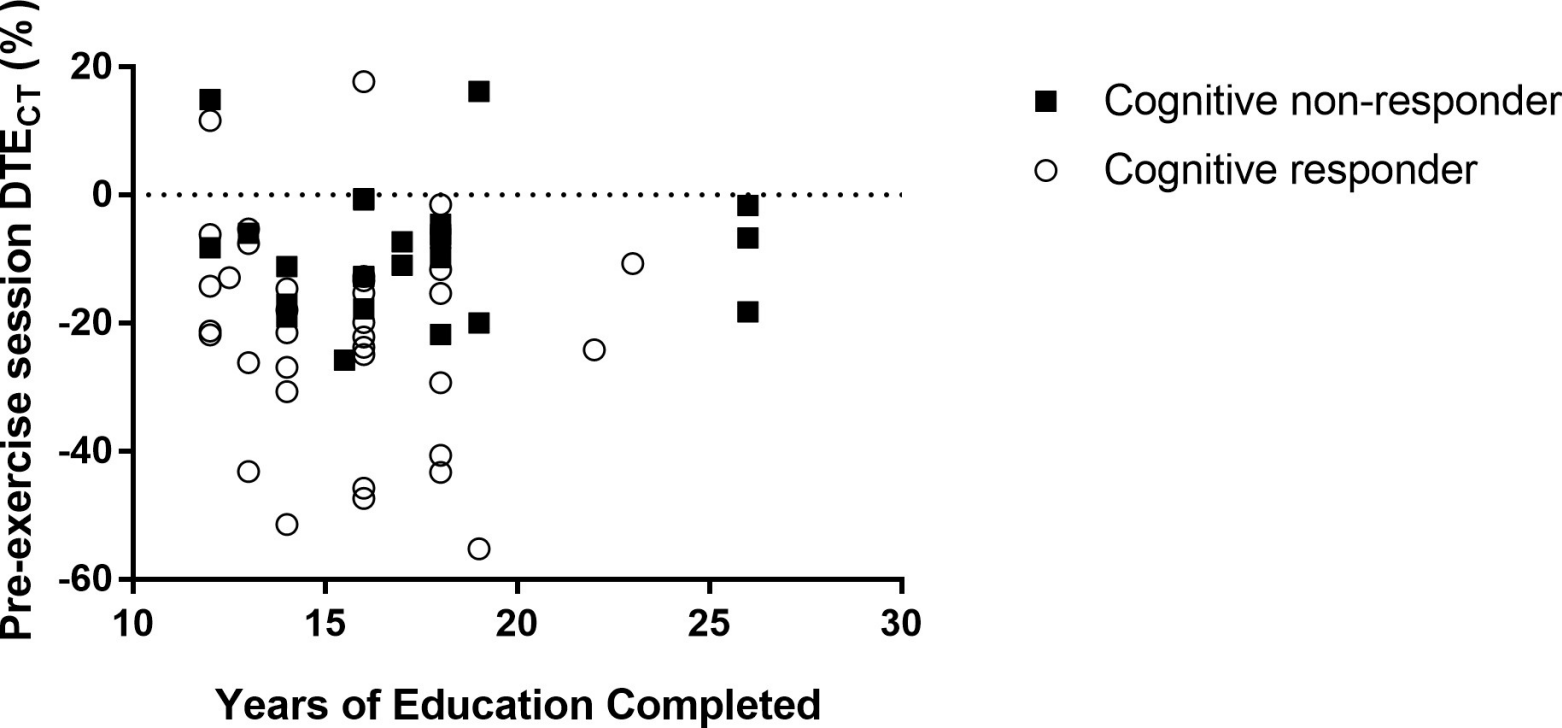
The relationship between years of education completed and DTE_CT_ measured during pre-exercise session testing. Subgroups indicate how dual-task effects on cognition changed after the exercise session, relative to pre-exercise session values.

**Fig 3:**
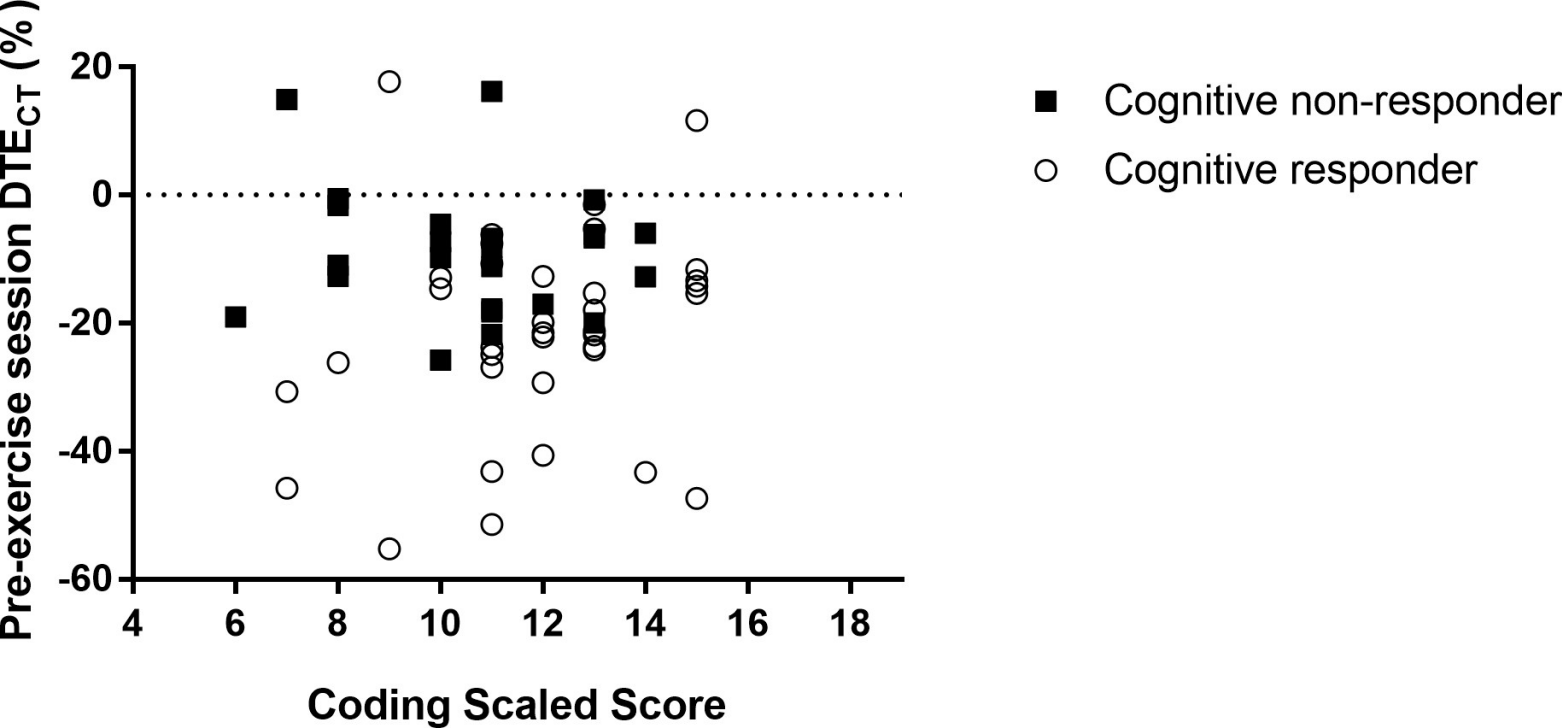
The relationship between Coding scaled scores and DTE_CT_ measured during pre-exercise session testing. Subgroups indicate how dual-task effects on cognition changed after the exercise session, relative to pre-exercise session values.

While it is possible that no differences were observed between the groups for the above-mentioned reasons, individuals within each group were also variable in how they responded to the intervention, likely contributing to the lack of group differences. To better understand this interindividual variability, the secondary purpose of this study was to analyze the baseline differences between those who reduced their dual-task costs on gait or cognition and those who did not, from pre- to post-training testing. Although there were no differences between gait responders and non-responders, there were differences between cognitive responders and non-responders. Specifically, the 36 cognitive responders completed an average of 1.8 fewer years of formal education and earned a Coding score that was 1.3 scaled points higher than the 24 non-responders. Responders completed an average of 15.5 years and non-responders completed an average of 17.3 years, which is not a large disparity, but the number of years roughly equate to earning a Bachelor’s degree (i.e., 16 years of education) and a 1-year Master’s degree (i.e., 17 years of education), which could be indicative of different levels of education achieved. More specifically, it appears that non-responders were more likely to pursue a post-graduate degree than responders. This distinction is significant because, although all individuals typically exhibit similar rates of cognitive decline, individuals with higher levels of formal education have been shown to start adulthood with better cognitive functioning and higher levels of cognitive reserve and thus take longer to decline, as they age, below a significant functional threshold than do individuals with lower levels of formal education [75]. While this relationship at first seems counterintuitive, in looking at Fig 2, it becomes apparent that non-responders typically exhibited dual-task benefits or smaller dual-task costs on cognitive throughput, relative to responders, during the pre-exercise session testing. Therefore, it could be inferred that those with higher levels of formal education did in fact exhibit higher levels of cognitive reserve, resulting in less dual-task costs on cognition during pre-exercise session testing and thus may not have had as much room to improve their dual-task effects on cognitive throughput as did the responders, who generally exhibited greater dual-task costs during pre-testing. These results are in agreement with those of Bamidis et al. [76] who determined that individuals who exhibit a higher baseline performance are less likely to increase their cognitive performance after an intervention due to a potential ceiling effect, relative to individuals who exhibited a lower baseline performance. Additionally, the groups differed in their information processing speed, as defined by the Coding subtest, scoring an average difference of 1.3 scaled points. While visuospatial reasoning and reaction time were important to the VRTT training and pre-/post-cognitive outcome measure, information processing speed is also an important component of a reaction time test like the clock task. Perhaps the responders, with their greater initial dual-task costs on cognitive throughput and faster information processing speed (Fig 3), were able to make faster gains with training. Of interest, processing speed is less related to acquired knowledge and education level than some other cognitive functions and has been shown to be more the result of age-related neurobiological degeneration [75, 77]. Therefore, it appears that both higher cognitive functioning at early adulthood and age-related declines in cognitive function impact the ability of older adults to reduce the DTE on cognition with acute training. In future studies, it would be interesting to see if both of these aspects of cognitive functioning maintain their influence on training outcomes during more long-term training.

### Limitations

This study is limited by a few factors. First, the short duration of the study limited the ability to identify potential differences between the experimental and control groups on dual-task performances because both interventions were similarly active, as previously noted. However, this study was designed to be a proof-of-concept study to determine if VRTT could result in acute improvements, which it did, regardless of the fact that it did not differ from the CTT in its acute effects. Future research should examine the long-term effects of these two interventions after an 8–12 week intervention. Second, some individuals may catch on more quickly and thus benefit more from VTT than others after a single session. With a larger study sample, we could have separated the VRTT group into two subgroups: those who acclimated quickly to interacting with the virtual reality and those who needed more consistent instruction throughout early training. Subsequent comparisons of the CTT group and the two VRTT subgroups would have allowed us to evaluate 1) conventional versus 2) quick acclimations to virtual reality versus 3) more instructions needed for virtual reality training. Third, subtle differences between the visuospatial training and the visuospatial outcome measure may have limited transfer of the learning during training to improve post-exercise testing. Greater transfer of learning may have been evident in the VTT group, relative to the CTT group, with the use of a cognitively-demanding motor task, like stepping over obstacles, as an outcome measure. Future research will include an analysis of stepping over obstacles as an outcome measure. Finally, this study enrolled older adults who had not fallen in the last year to ensure generalizability of the results to a broad population of older adults. While enrolling older adults prone to falling into the study could have resulted in interesting findings, it also could have made interpretation of the findings difficult because the causes of falls are multifactorial (e.g., vestibular issues, muscle weakness, vision impairments, etc.). Future research should explore the efficacy of VRTT in older adults prone to falling for various reasons.

## Conclusions

The results of this study provide evidence that there are no differences in the acute effects on gait and cognition after a single session of VRTT or CTT. Instead, similar improvements in gait and cognition during both single- and dual-task performances, as well as reductions in the dual-task effects on both gait and cognition, were observed following the VRTT and CTT training sessions. Therefore, it may be inferred from these results that older adults should be able to choose from either virtual reality treadmill training or more conventional one-on-one training using a treadmill, as they are likely to benefit from either. The results of the study also provide evidence that level of formal education completed and information processing speed can impact whether or not older adults reduce the dual-task effects on cognition after a single session of exercise training. The differential impact of virtual reality versus conventional training and the impact of cognitive functioning on training outcomes should be further explored during more long-term training.

## Supporting information

S1 Data (XLSX)
